# Psychometric properties of the Chinese version of the fatigue scale-adolescent

**DOI:** 10.1186/s12885-015-1945-y

**Published:** 2015-11-27

**Authors:** Ka Yan Ho, William H. C. Li, Ka Wai Katherine Lam, S. Y. Chui, Chi-Fung Godfrey Chan

**Affiliations:** 1 School of Nursing, The University of Hong Kong, 4/F, William MW Mong Block, No 21 Sassoon Road, Pokfulam, Hong Kong; 2 Department of Adolescent Medicine, Queen Mary Hospital, Room 115, New Clinical Building, 102 Pokfulam Road, Hong Kong, Hong Kong

**Keywords:** Adolescent, Cancer, Chinese, Confirmatory factor analysis, Fatigue, Pediatric oncology

## Abstract

**Background:**

The availability of a valid and reliable instrument that accurately assesses the level of fatigue among adolescent cancer survivors is crucial before any appropriate interventions to reduce their fatigue can be appropriately planned and evaluated. The study aimed to test the psychometric properties of the Chinese version of the Fatigue Scale for Adolescents. In particular, confirmatory factor analysis was conducted to examine its factorial structure.

**Methods:**

A cross-sectional study was employed. Adolescents (13- to 18-year-olds) who had survived cancer and attended medical follow-up at the outpatient clinic in Hong Kong were invited to participate. The internal consistency, content validity and construct validity and test-retest reliability of the Chinese version of the Fatigue Scale for Adolescents were assessed.

**Results:**

The content validity index was 0.92. There was a strong positive correlation between adolescents’ levels of fatigue and depressive symptoms (*r* = 0.53) and a strong negative correlation between adolescents’ levels of fatigue and quality of life (*r* = –0.58). The mean levels of fatigue of the survivors group was significantly lower than that of those still receiving treatment in hospital, but significantly higher than that of their healthy counterparts. Confirmatory factor analysis indicated that there were 4 factors underlying the Chinese version of the Cancer Module.

**Conclusions:**

The findings of the study add further evidence that the Chinese version of the Fatigue Scale for Adolescents (12-item) can be used as a reliable and valid tool in assessing cancer-related fatigue among Hong Kong Chinese adolescents who have survived cancer.

## Backgrounds

Notwithstanding a significant decrease in cancer mortality rates as a result of the advances in the field, cancer and its treatment may have short- and long-term adverse effects on the physical and psychological well-being of adolescent cancer patients [1, 2] and survivors [3, 4]. The accumulated adverse effects of surgical intervention, chemotherapy and/or radiotherapy may damage normal body tissue and impair physical fitness [3], leading to reduced cardio-respiratory function, decreased muscle strength, and fatigue [5]. Such complications may continue for months or even years after the completion of therapy [1].

Of all the adverse effects of treatment, cancer-related fatigue is the most common physical concern reported by adolescents hospitalized with cancer [6–8] and those surviving the disease [9, 10]. Previous studies indicate that about 70 % of patients being treated and up to 30 % of survivors report feelings of fatigue [11, 12]. Cancer-related fatigue is a clinical symptom generally defined as a person’s subjective feeling of persistent tiredness and exhaustion that cannot be relieved by rest [13]. The fatigue can be distressing for adolescents, as it may exacerbate other unpleasant symptoms such as nausea, dyspnea and pain [14]. There is also some evidence that fatigue is correlated with depression and sleep disturbance [15]. Additionally, cancer-related fatigue can severely limit the capacity to perform ordinary daily activities such as reading and studying, watching television, playing computer/electronic games, and particularly outdoor leisure activities, substantially impairing quality of life [9, 16, 17]. Nevertheless, despite its detrimental effects on the physical and psychological well-being of cancer patients and survivors, cancer-related fatigue is often overlooked or underestimated by healthcare professionals, compared with other treatment-related symptoms such as pain, nausea and vomiting [18, 19].

It has been well documented that regular exercise can help ameliorate adverse cancer treatment-related effects, in particular fatigue [20, 21]. Nevertheless, there is growing concern about declining levels of physical activity in childhood cancer survivors [11]. A cross-sectional study [9] assessing physical activity and the factors affecting regular exercise among 128 young Hong Kong Chinese cancer survivors revealed that their current physical activity levels were markedly reduced when compared with the pre-morbid situation. Many reported that fatigue after remission prevented them from engaging in regular physical activity [9]. There is scientific evidence that physical inactivity induces muscle catabolism and atrophy [5] and cardiovascular diseases [11], which may lead to a further increase in fatigue and a decrease in the functional capacity of cancer patients. It is vital, therefore, for healthcare professionals to develop and evaluate appropriate interventions that can help reduce fatigue among young cancer patients and survivors, and increase their awareness of the importance of regular physical activity. First, however, the availability of a valid and reliable instrument that accurately documents and evaluates the level of fatigue among young cancer patients and survivors is crucial before any appropriate interventions can be appropriately planned and evaluated.

A review of the literature reveals that there are different instruments used to measure fatigue for adolescent and adult cancer survivors [22–25]. One promising scale for measuring fatigue in adolescents is the Fatigue Scale for Adolescents (FS-A), which has been widely used in clinical settings and research [24]. The FS-A was developed by Hinds et al. [25] based on a conceptual model derived from a qualitative study about fatigue during treatment of cancer in adolescents. The psychometric properties of the FS-A were tested [25], with the results demonstrating adequate internal consistency and appropriate construct validity using the known-groups method (anemic vs non-anemic). The factorial structure of the FS-A was examined using exploratory factor analysis [25], and the results showed four underlying factors: (1) cognitive and physical weariness, (2) added effort and assistance needed to do usual activities, (3) needing rest and feeling angry and (4) avoiding social activities. Since then, a number of studies have been carried out to document the level of cancer–related fatigue among adolescents using the FS-A, and to evaluate its psychometric properties [26–28]. All such studies have generally supported the FS-A as a suitable tool for evaluating fatigue or the effects of an intervention on fatigue in a population of adolescents with cancer. Nevertheless, although the FS-A has been translated into Chinese and used with adolescents in Taiwan [27], it has not been used with Hong Kong Chinese adolescents. Because of cultural differences between Western countries, Taiwan and Hong Kong, some concepts or items in the original instrument may be inappropriate for adolescents of another culture. As a result, the findings may be inaccurate. Moreover, the FS-A was primarily developed to measure adolescents’ cancer-related fatigue during treatment, when they experienced the highest levels of fatigue. It is therefore unclear whether the FS-A can also be used to assess the fatigue of adolescents who have completed cancer treatment. Most importantly, because of the small sample size used in previous studies [25–28], confirmatory factor analysis was not performed to test the hypothesized configuration of the factor structure or measurement model for the FS-A. Consequently, whether the factor structure of the Chinese FS-A version is congruent with the findings of previous exploratory factor analysis cannot be confirmed. Given these issues, before using the Chinese version of the FS-A for adolescent cancer survivors in the Hong Kong Chinese context, it is necessary to evaluate both linguistic and cultural equivalence. This study aimed to test the psychometric properties of the Chinese version of the Fatigue Scale for Adolescents. In particular, it examined the factorial structure of the FS-A using confirmatory factor analysis.

## Methods

### Study design and sample

A cross-sectional study was used. Adolescents who had survived cancer and attended medical follow-up at the outpatient clinic of a public acute-care hospital in Hong Kong were invited to participate. Hong Kong Chinese adolescents, who met the inclusion criteria for the study, were invited to participate. The inclusion criteria were: (1) cancer survivors who had completed treatment at least 6 months previously, (2) aged between 13 and 18, and (3) able to speak Cantonese and read Chinese. We excluded adolescents with evidence of recurrence or second malignancies and those with cognitive and learning problems identified from their medical records.

There are no clear rules as to sample size for factor analysis, and there is little agreement among researchers regarding how large a sample should be. Gorsuch [29] claims that there should be at least five subjects per variable, and a minimum of 200 subjects is recommended for confirmatory factor analysis. With all this in mind, a convenience sample of 200 subjects was recruited during a 24-month period from 2012 to 2014.

To test construct validity using the known-groups method, another two similar age groups of 50 patients receiving treatment for cancer in a pediatric oncology unit and 50 healthy counterparts, members of an integrated child and youth service center, were invited to join the study.

### Study instruments

#### The Fatigue Scale-Adolescent Instrument (FS-A)

The FS-A is designed to measure fatigue in adolescents with cancer aged 13 to 18 years [25]. It consists of 14 items. Responses are rated using a 5-point Likert scale ranging from 1 to 5 (1 = not at all; 2 = a little; 3 = some; 4 = quite a bit; 5 = a lot). All items were coded in the same direction with the exception of item 9 “Able to do usual activities,” was reverse-coded. Total possible scores range from 14 to 70. Higher scores indicate higher levels of fatigue. The FS-A has been used in Taiwanese adolescents [27]. The results of psychometric tests showed acceptable internal consistency (α = .89) and moderate-to-high content validity (content validity index ranges from 87 to 100 %).

### Center for Epidemiologic Studies Depression Scale for Children (CES-DC)

The CES-DC comprises 20 standardized items to evaluate depressive symptoms. All items are evaluated on a 4-point Likert scale in relation to their incidence during the previous week, and are scored from 0 to 3 (0 = not at all, 1 = a little, 2 = some, 3 = a lot); total possible scores thus range from 0 to 60, with higher scores indicating greater number of symptoms. The psychometric properties of the Chinese version of the CES-DC have been empirically tested [30] with internal consistency found to have an α coefficient of .82, construct validity showed a convergent validity of *r* = 0.63, *p* < .01 and discriminant validity of *r* = – 0.52, *p* < .01.

### The Chinese version of the Pediatric Quality of Life Inventory (PedsQL)

The PedsQL was used to measure the participants’ quality of life. It comprises 23 items to rate how often they had experienced problems over the past month on a scale from 0 to 4. Higher scores indicate better quality of life. The psychometric properties of the Chinese version of the PedsQL have been empirically tested [31], with internal consistency found to have an alpha coefficient of.86, and test–retest reliability ranging from *r* = 0.62 to *r* = 0.8.

### Translation process

The FS-A was translated according to the recommendations suggested by Bracken and Barona [32]. During the process, a researcher would translate the FS-A from English to Chinese. Another bilingual translator who blinded to the FS-A was then asked to complete the back-translation. To ensure the meaning of each item was kept, a comparison was made between the original and re-translated English versions. Disagreements were discussed and agreed upon by both the researcher and the back-translator.

### Data collection methods

Approval for the study was obtained from the Institutional Review Board of the University of Hong Kong/ Hospital Authority Hong Kong West Cluster. Written consent was then obtained from all parents after they were told the purpose of the study. They were given the option of allowing or refusing their child’s involvement in the study. According to the Hong Kong Institutional Review Board regulations, participant aged 18 years and under requires consent from a parent or guardian. The adolescents were also invited to put their names on a special assent form and told that their participation was voluntary.

After obtaining demographic data from all participants, the group of adolescents who had survived cancer (*n* =200) were asked to complete the Chinese versions of the FS-A, CES-DC and PedsQL, while those receiving treatment (*n* = 50) and their healthy counterparts (*n* = 50) only responded to the FS-A.

### Data analysis

The Chinese version of the FS-A was subjected to equivalence testing of its semantic and content dimensions. Semantic equivalence implies that each item remains conceptually and idiomatically the same after translation; content equivalence implies that each item in the instrument has consistent cultural relevance and an appropriate sample of items for the construct being measured [33]. To establish the semantic and content equivalence of the Chinese version of the FS-A, a panel of experts was set up, which included a pediatric oncology nurse specialist, two pediatric oncology researchers and three lecturers working at a local university, all of whom were bilingual and had experience of translating and validating instruments.

### Semantic equivalence

The panel of experts was asked to rate the equivalence of translation between each item of the original English and Chinese versions of the FS-A using a 4-point rating scale (from 1 = not equivalent to 4 = most equivalent). Based on their responses, an equivalence rate (the percentage of the total items rated by the experts as either 3 or 4) was calculated. Any item deemed not equivalent by receiving a rating 1 or 2 by more than 20 % of respondents would be amended.

### Content equivalence

Content equivalence of the Chinese version of the FS-A was established by the expert panel. They were asked to rate the relevancy of each item to the concept of cancer-related fatigue for adolescent cancer survivors on a 4-point scale (from 1 = not relevant to 4 = very relevant). The Content Validity Index (CVI) is the percentage of the total items rated as either 3 or 4. A CVI score of 90 % or higher is generally considered to indicate good content validity [34].

### Construct validity testing

To assess the known-groups validity of the Chinese version of the FS-A, a one-way between-groups analysis of variance with post-hoc tests was conducted to compare the levels of fatigue among 50 adolescent participants who had survived cancer (randomly selected from the pool; *n* = 200), 50 receiving treatment and 50 healthy counterparts. It was expected that the survivor group would report lower levels of fatigue than those still in hospital, but higher levels than the healthy group.

Convergent validity was established by finding correlations between scores on the Chinese versions of the FS-A and CES-DC using the Pearson product–moment correlation coefficient. Previous studies indicated that people with higher levels of fatigue would report more depressive symptoms [15, 27]. We hypothesized that there would be a positive correlation between the FS-A and CES-DC scores.

Discriminant validity was estimated by examining the correlation between scores on the FS-A and PedsQL. There is some evidence that cancer-related fatigue would adversely affect the quality of life among adolescents who had survived cancer [9, 16, 17]. We hypothesized that there would be a negative correlation between the FS-A and PedsQL scores.

To allow more precise testing of the configuration of the factor structures of the Chinese version of the FS-A and to examine whether the proposed factor structures (4-factor model) adequately fitted the data, CFA was carried out using LISREL version 8.8 for Windows (Scientific Software International Inc, Lincolnwood, Illinois). The parameters were estimated by the generally weighted least-squares method, using asymptotic covariance matrixes. The overall fit of the data model with the scale was then examined by goodness-of-fit indices, including the *χ*^2^/degrees of freedom (*df*) ratio, root mean square error of approximation (RMSEA), comparative fix index, and goodness-of-fit index. The *χ*^2^/*df* ratio is a measure of global fit. An *χ*^2^/*df* value between 1 and 5 indicates a good fit [35]. The RMSEA is an indication of model fit and is based on the population discrepancy function, which is a standardized measure of error of approximation [36]. In general, RMSEA values of less than 0.05 indicate superior model fit [37]. The goodness-of-fit index is a measure of global fit between a theoretical model and the data, where a value of 0.90 or higher is considered to indicate a good model-data fit [38]. The comparative fix index is the indicator of how much better the model fits compared with an independence model. These measures vary from 0 to 1.00, a value of 0.95 or higher indicating a good fit [39].

### Reliability testing

Internal consistency reliability of the Chinese version of the FS-A was assessed by calculating Cronbach’s alpha. To examine the stability of the FS-A, 20 % of the survivors group (*n* =40) were randomly selected to respond to the FS-A again after 2 weeks, via telephone follow-up. The intraclass correlation coefficient (ICC) was used to estimate the test–retest reliability coefficient.

## Results

### Participant demographics

The demographic data are shown in Table 1. There were similar numbers of boys and girls in the survivor group. About half of them had been diagnosed with leukemia and lymphoma (74.0 %). Most (86.5 %) had completed their entire medical treatment within 5 years, with only 27 continuing for longer. In addition, the results show that three groups were similar with respect to the age and gender of the adolescents.

**Table 1:** Demographic Characteristics of the Participants (*N* =300)

	*n* (%)				
	Childhood cancer survivors (*n* = 200)	Cancer children (*n* = 50)	Healthy Children (*n* = 50)	*x* ^2^	*p*
Age (years)				6.8	0.9 ^ns^
13	20 (10.0)	6 (12.0)	8 (16.0)		
14	28 (14.0)	7 (14.0)	7 (14.0)		
15	37 (18.5)	11 (22.0)	10 (20.0)		
16	33 (16.5)	7 (14.0)	8 (16.0)		
17	39 (19.5)	10 (20.0)	6 (12.0)		
18	43 (21.5)	9 (18.0)	11 (22.0)		
Sex				0.3	0.9 ^ns^
Male	108 (54.0)	28 (56.0)	26 (52.0)		
Female	92 (46.0)	22 (44.0)	24 (48.0)		
Parents’ Educational Attainment				1.0	1.0 ^ns^
Primary school or below	32 (16.0)	6 (12.0)	7 (14.0)		
Lower secondary school	63 (31.5)	15 (30.0)	14 (28.0)		
Upper secondary school	71 (35.5)	19 (38.0)	20 (40.0)		
Tertiary education	34 (17.0)	10 (20.0)	9 (18.0)		
Diagnosis				6.5	0.3 ^ns^
Leukemia	91 (45.5)	19 (38.0)	-		
Lymphoma	57 (28.5)	12 (24.0)	-		
Brain tumor	33 (16.5)	8 (16.0)	-		
Osteosarcoma	9 (4.5)	4 (8.0)	-		
Kidney tumor	4 (2.0)	3 (6.0)	-		
Germ-cell tumor	6 (3.0)	4 (8.0)	-		
Treatment received				3.6	1.0^ns^
Surgery	23 (11.5)	5 (10.0)	-		
Chemotherapy	90 (45.0)	22 (44.0)	-		
Bone Marrow Transplant	22 (11.0)	5 (10.0)	-		
Mixed method:					
Chemotherapy and radiotherapy	12 (6.0)	3 (6.0)	-		
Surgery and chemotherapy	19 (9.5)	5 (10.0)	-		
Chemotherapy and bone marrow transplantation	23 (11.5)	7 (14.0)	-		
Radiotherapy and surgery	11 (5.5)	3 (6.0)	-		
Time since treatment completed					
6 - 12 months	39 (19.5)	-	-		
13 – 24 months	37 (18.5)	-	-		
25 – 36 months	33 (16.5)	-	-		
37 – 48 months	31 (15.5)	-	-		
48 – 60 months	33 (16.5)	-	-		
>60 months	27 (13.5)	-	-		

*ns* Not significant at *P* >0.05

### Validity

#### Semantic equivalence

The average equivalence rate was 94 % (range from 92 to 96 %), indicating that each item of the Chinese version of the FS-A remained conceptually and idiomatically the same as in the English version.

### Content equivalence

The content validity index (CVI) was 82 % (range 17 to 100 %). The majority of items, with the exception of numbers 6 and 10, were rated as quite or very relevant, indicating that the content of most FS-A items reflected the underlying construct. Omitting items 6 and 10, the CVI was re-calculated at .92 (92 %; range 83 to 100 %), indicating the valid content validity of the Chinese version.

### Construct validity

The results of one-way between-groups analysis of variance with post-hoc tests on the levels of fatigue among adolescents who had survived cancer, receiving treatment and healthy counterparts are shown in Table 2. The results showed that the mean FS-A score of the survivors group was significantly lower than that of those still receiving treatment in hospital, but significantly higher than that of their healthy counterparts. The known-groups validity was supported.

**Table 2:** The results of ANOVA on the levels of fatigue among the three groups

	Mean (SD)			G1 Vs G2		G1 Vs G3		G2 Vs G3	
	G1	G 2	G 3	Mean Difference	*p*-value	Mean Difference	*p*-value	Mean Difference	*p*-value
Levels of fatigue	28.6 (3.7)	31.3 (5.2)	22.1 (4.8)	−2.7	0.03	6.5	0.000^a^	9.2	0.000^a^

^a^Significant at *p* <0.005

*SD* standard deviation, *G1* adolescents who had survived cancer, *G2* adolescents receiving treatment, *G3* healthy counterparts; with each group contained 50 subjects

The inter-relationships among scores on the FS-A, CES-DC and PedsQL were examined. Correlation coefficients of 0.10 to 0.29, 0.30 to 0.49 and 0.50 to 1.0 are typically interpreted as small, medium and large, respectively [40]. There was a strong positive correlation between scores on the FS-A and CES-DC (*r* = 0.53, *n* = 200, *P* < 0 .01), indicating that adolescents with higher levels of fatigue were associated with more depressive symptoms. In addition, there was a strong negative correlation between scores on the FS-A and PedsQL (*r* = −0.58, *n* = 200, *P* < 0 .01), indicating that higher levels of fatigue were to be associated with lower quality of life. The results support the discriminant validity of the FS-A.

### Confirmatory factor analysis

**Fig. 1:**
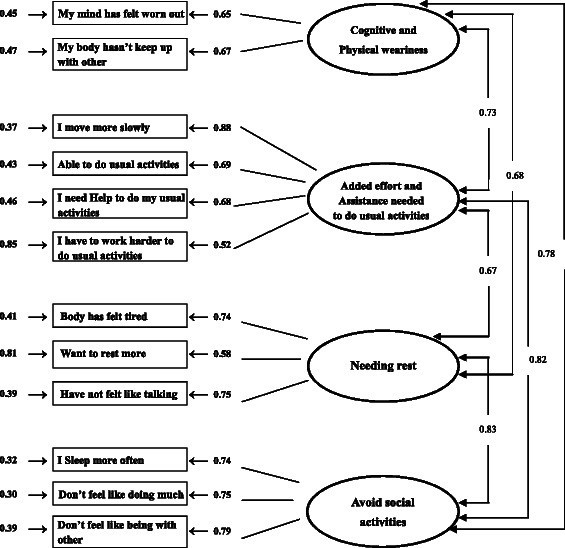
Confirmatory factor analysis model for the Chinese version of the Fatigue Scale-Adolescent (FS-A)

The overall fits of the 14-, 13- and 12-item of the Chinese version of the FS-A was tested by a variety of fit indices based on the proposed 4-factor model. The results revealed that the 12-item 4-factor model was the best fit across all fit indices (Table 3). The parameter estimates of this 4-factor model (12-item) are shown in Fig. 1. This shows that all correlation matrices were less than 1.00 and were positive-definite, indicating that the parameter estimated was reasonable. The factor loading for each observed variable was high, ranging from 0.48 to 0.90. The *t*-values of all variables were greater than 2.00, suggesting statistically significant loadings. The standard errors ranged from 0.28 to 0.92, indicating that all the parameters were accurately estimated [37].

**Table 3:** Fit Statistics for the 14-, 13- and 12-item four factor-structure model of the Chinese version of the Fatigue Scale for Adolescents (FS-A)

Factor model	*χ*^2^/df	CFI	GFI	RMSEA
Chinese version of the FS-A				
14-item 4-factor model	4.87	0.90	0.89	0.06
13-item 4-factor model	4.52	0.91	0.89	0.06
12-item 4-factor model	4.06	0.92	0.91	0.05

Abbreviations: *χ*^2^/df, Relative chi-square; CFI, Comparative fix index; GFI Goodness-of-fit index; RMSEA, Root Mean Square Error of Approximation

Footnotes

Confirmatory factor analysis (CFA) was carried out using LISREL version 8.8 for Windows. Acceptable overall fit of each model was evaluated using the following indices:

### Reliability

The test-retest reliability coefficient of the Chinese version of the FS-A at a 2-week interval was 0.85 (ICC-value), indicating a reliability of 0.80 or higher, which is acceptable for an instrument to be used in research [41]. The internal consistency (14 items) was found to have a Cronbach’s alpha of 0.86. The corrected item-total correlation values ranged from 0.10 to 0.71. Most items correlated with the total score, with the exception of items 6 and 10, where the corrected item-total correlation values were 0.10 and 0.23, respectively, indicating that the items are measuring something different from the scale as a whole. After a thorough discussion in the expert panel, all members agreed to delete items 6 and 10, when the Cronbach alpha coefficient was re-calculated at 0.89.

## Discussion

The study aimed to test empirically the psychometric properties of the Chinese version of the FS-A that had not so far been tested in the Hong Kong Chinese context. In particular, CFA was performed to examine whether the factor structures proposed by the previous EFA adequately fit the data.

Although the psychometric properties of the FS-A have been tested in the United States and Taiwan, it may be psychometrically inappropriate and even problematic to apply this tool to a different cultural group, such as Hong Kong Chinese adolescents. Because the meaning of the construct the instrument is designed to measure may differ from one culture to other, it is of paramount importance to ensure cultural equivalence before using a translated version of the scale in the Hong Kong Chinese context. The cultural context in question is one where Chinese parents have high expectations of their children, and most parents and schools place very considerable emphasis on academic achievement [42], probably because of the common attitude, held by parents and by most of their children, that academic achievement is related to a better career and a brighter future. It is therefore valued above other forms of achievement in Chinese society. Given this cultural context, Hong Kong children are under tremendous pressure to cope with a heavy homework load and generally to excel in academic performance [42]. Therefore, when responding to item 6, ‘It’s harder to keep up with school work’, 37.5 % of the adolescents answered ‘some’ and 51.5 % answered ‘quite a bit’ or ‘a lot.’ Moreover, apart from fatigue, poor concentration and a decreased attention span were also common physical concerns reported by survivors causing them learning difficulties at school [43]. Additionally, a previous study [9] revealed that many cancer survivors were anxious about their academic performance, which they claimed was significantly affected by the cancer and its treatment. As a result of suspension from school during the course of treatment, they had to make extra efforts to keep up with school work. Consequently, it is unclear why exactly they had to work harder to keep up at school.

Another example of cultural incongruence is the well-documented notion that Hong Kong Chinese people are influenced by Confucian philosophy [2, 44], which emphasizes balance and harmony achieved through the notions of *chung* and *yung* in everyday living [9]. Under the influence of this philosophy [2], many adolescents surviving cancer might prefer to keep themselves calm and in control in dealing with their situation. When responding to item 10, ‘I have felt angry’, the majority (83 %) answered ‘not at all.’ In fact, evaluating the content equivalence of the Chinese FS-A version, items 6 and 10 were found not to be equivalent by the panel. Additionally, the results of item-total correlation indicated these two items were measuring different psychological constructs. Given these issues, it was decided that items 6 and 10 should be deleted from the Chinese version.

Construct validity was confirmed by the known-groups method. It is understandable that adolescents under treatment in hospital should report higher levels of fatigue than those who had survived cancer, and that overall healthy adolescents should report the least fatigue. The results demonstrated that the Chinese version of the FS-A was able to differentiate levels of fatigue among various groups of adolescents, and thus could serve as a valid tool to assess fatigue among adolescents who had completed cancer treatment.

Construct validity was supported by correlations observed between scores on the Chinese versions of the FS-A and CES-DC. The findings were consistent with those of previous studies showing that adolescents with higher levels of fatigue would present more depressive symptoms [15, 27]. The discriminant validity of the Chinese version of the FS-A was also supported. In accord with previous studies [9, 16, 17], the results revealed a strong negative correlation between scores on the Chinese versions of the FS-A and PedsQL, suggesting that the quality of life among adolescents would be adversely affect by cancer-related fatigue.

To test the instrument’s factor structure more precisely, confirmatory factor analysis was performed to evaluate whether the hypothesized four-factor structure of the Chinese FS-A version would fit the data adequately. Overall, the results of the evaluation fit were convincing, and confirmed that there was a very good fit between the hypothesized model [31] and the data. Most importantly, the results revealed that the 12-item 4-factor model was the best fit across all fit indices, provided further support of deleting items 6 and 10 from the Chinese version.

When compared with other instruments measuring fatigue among survivors of adult and childhood cancer from different ethnic groups [22, 23], the Chinese version of FS-A demonstrated adequate internal consistency, good content validity, appropriate convergent and discriminant validity, and excellent construct validity. Most importantly, all participants in this study were able to provide full responses to the questionnaire, without showing any particular difficulty in understanding the questions. Indeed, the Chinese version of FS-A is user-friendly in that it is easy to comprehend and quick to complete—it took 5 to 8 min for each adolescent to complete the questionnaire.

### Implications for practice

The results of the study add further evidence that adolescents surviving cancer but reporting higher levels of fatigue after remission exhibit more depressive symptoms and report a lower quality of life. There is thus a compelling need for healthcare professionals to provide long-term follow-up for those adolescents who have survived cancer, in particular to monitor and document their levels of fatigue and the complications arising from this adverse effect of treatment [45]. Another important implication for practice is that regular exercise has been shown to improve fitness and physical functioning, increase muscle strength, and reduce cancer-related fatigue. It is therefore crucial for healthcare professionals to develop interventions that can help reduce cancer-related fatigue, and to promote the adoption of regular physical activity among adolescents surviving cancer. The validated instrument may be used in assessing Chinese adolescent survivors for the presence of fatigue and evaluating the effectiveness of interventions for reducing fatigue.

## Conclusion

This study addressed a gap in the literature by examining the psychometric properties of the Chinese version of the FS-A. The results of CFA confirmed that the factorial structure of this Chinese version was congruent with the proposed four-factor model of the original English version. Most importantly, the findings of the study add further evidence that the Chinese version of the FS-A (12-item) can be used as a reliable and valid tool in assessing cancer-related fatigue among Hong Kong Chinese adolescents who have survived cancer.
